# BB-10010/MIP-1 alpha in vivo maintains haemopoietic recovery following repeated cycles of sublethal irradiation.

**DOI:** 10.1038/bjc.1996.483

**Published:** 1996-10

**Authors:** B. I. Lord, E. Marshall, L. B. Woolford, M. G. Hunter

**Affiliations:** CRC Department of Experimental Haematology, Paterson Institute for Cancer Research, Manchester, UK.

## Abstract

Macrophage inflammatory protein-1 alpha (MIP-1 alpha) is an inhibitor of stem cell proliferation affording protection against damage from agents that express their cytotoxicity specifically in the DNA synthesis phase of the cell cycle. Its ability also to modify the self-renewal capacity of the regenerating cells is now shown to improve and maintain haemopoietic recovery following therapy (sublethal irradiation) whose cytotoxic damage is not limited solely to the DNA-S phase of this cycle. Such non-cell cycle-active cytotoxic agents are used clinically in repeated treatment regimens, which are often limited or terminated because of accumulating haemopoietic damage. BB-10010, a non-aggregating variant of MIP-1 alpha, was administered as a continuous dose (1600 micrograms kg-1 24 h-1) via a subcutaneously implanted pump over a period of 7 days. A dose of 4.5 Gy total-body gamma-rays was given 3-4 h after implantation. Day 8 and 12 spleen colony-forming units (CFU-S) were assayed on days 1, 7 and 14 after irradiation. This cycle of treatment was repeated four times (total 56 days), and on day 14 of the last two cycles the marrow-repopulating ability (MRA) was also measured. In the control bone marrow (no BB-10010) CFU-S fell to < 1% of normal within 1 day of irradiation and recovered to 40% at 14 days. Repeated treatments increased the level of damage, and after four cycles CFU-S recovered to only 10% of normal. BB-10010 afforded little benefit in the first treatment cycle, but by the end of the fourth cycle CFU-S still recovered to 35% of normal. MRA was reduced to 7% of normal by the irradiation protocol-about half that maintained by BB-10010 protection. We conclude that BB-10010 (MIP-1 alpha) reduces the degree of accumulated haemopoietic stem cell damage following repeated non-cell cycle-specific cytotoxic insults-a principle which should be valuable in repeated clinical cytotoxic therapy regimens.


British Journal of Cancer (1996) 74, 1017–1022
© 1996 Stockton Press All rights reserved 0007–0920/96 $12.00

### BB-10010/MIP-1α *in vivo* maintains haemopoietic recovery following repeated cycles of sublethal irradiation

BI Lord[1], E Marshall[2], LB Woolford[1] and MG Hunter[3]

[1]*CRC Department of Experimental Haematology, Paterson Institute for Cancer Research, and* [2]*Department of Medical Oncology, Christie Hospital NHS Trust, Wilmslow Road, Manchester M20 4BX, UK;* [3]*British Biotech Pharmaceuticals Ltd, Watlington Road, Oxford OX4 5LY, UK.*

**Summary** Macrophage inflammatory protein-1α (MIP-1α) is an inhibitor of stem cell proliferation affording protection against damage from agents that express their cytotoxicity specifically in the DNA synthesis phase of the cell cycle. Its ability also to modify the self-renewal capacity of the regenerating cells is now shown to improve and maintain haemopoietic recovery following therapy (sublethal irradiation) whose cytotoxic damage is not limited solely to the DNA-S phase of this cycle. Such non-cell cycle-active cytotoxic agents are used clinically in repeated treatment regimens, which are often limited or terminated because of accumulating haemopoietic damage. BB-10010, a non-aggregating variant of MIP-1α, was administered as a continuous dose (1600 $\mu g\ kg^{-1}\ 24\ h^{-1}$) via a subcutaneously implanted pump over a period of 7 days. A dose of 4.5 Gy total-body γ-rays was given 3–4 h after implantation. Day 8 and 12 spleen colony-forming units (CFU-S) were assayed on days 1, 7 and 14 after irradiation. This cycle of treatment was repeated four times (total 56 days), and on day 14 of the last two cycles the marrow-repopulating ability (MRA) was also measured. In the control bone marrow (no BB-10010) CFU-S fell to <1% of normal within 1 day of irradiation and recovered to 40% at 14 days. Repeated treatments increased the level of damage, and after four cycles CFU-S recovered to only 10% of normal. BB-10010 afforded little benefit in the first treatment cycle, but by the end of the fourth cycle CFU-S still recovered to 35% of normal. MRA was reduced to 7% of normal by the irradiation protocol – about half that maintained by BB-10010 protection. We conclude that BB-10010 (MIP-1α) reduces the degree of accumulated haemopoietic stem cell damage following repeated non-cell cycle-specific cytotoxic insults – a principle which should be valuable in repeated clinical cytotoxic therapy regimens.

**Keywords:** haemopoiesis; macrophage inflammatory protein-1α; stem cell inhibitor; stem cell protection; repeated cytotoxic treatment

Macrophage inflammatory protein-1α (MIP-1α) has been recognised as a haemopoietic stem cell proliferation inhibitor by its capactiy to protect multipotent progenitor cells from cytotoxic agents, which are effective against cells specifically in DNA synthesis, both *in vitro* and *in vivo* (Lord *et al.*, 1976; 1992 Wright and Lord, 1977; Graham *et al.*, 1990; Clements *et al.*, 1992; Dunlop *et al.*, 1992; Cooper *et al.*, 1994). In one series of experiments in which MIP-1α was given *in vivo* to protect haemopoietic spleen colony-forming units (CFU-S) (Till and McCulloch, 1962) from the effects of hydroxyurea (HU), the subsequent recovery rate of the (partially) protected population appeared to be higher than in the control (HU-treated) population (Lord *et al.*, 1992). This was complementary to an earlier observation that the partially purified inhibitor enhanced the generation of haemopoietic cells in long-term bone marrow cultures (Lord *et al.*, 1987). Furthermore, it led to the suggestion that, in addition to its capacity to block the progression of progenitor cells into DNA synthesis, MIP-1α had effects also on the self-renewal and differentiation capacity of the surviving multipotent progenitor cell population (Lord *et al.*, 1992). This enhanced rate of recovery of the CFU-S population has recently been confirmed and direct measurements showed the self-renewal capacity of the CFU-S population surviving HU treatment to be enhanced by MIP-1α given *in vivo* (Lord, 1995).

These observations together suggested that the clinical value of MIP-1α need not be limited to protection of haemopoietic stem cells from cytotoxic agents which specifically damage the DNA synthesis phase of the cell cycle. CFU-S populations depleted by non-cell cycle-specific treatments, but exposed to MIP-1α, might emerge with a higher self-renewal capacity, thus restocking the stem cell compartments more efficiently and making them less susceptible to the accumulating damage incurred by the repeated treatment cycles commonly used in chemotherapeutic regimens.

Repeated sublethal doses of irradiation result in a long-term accumulation of damage such that recovery of the CFU-S population is successively more impaired, i.e. the plateau level of CFU-S recovery is lower with each successive cycle of radiation (Hendry and Lajtha, 1972). Such damage is equivalent to that frequently encountered in chemotherapy programmes where successive cycles of treatment may have to be delayed or terminated after four or five cycles because of deteriorating marrow recovery. We have now used this radiation model as a form of repeated courses of cytotoxic treatments and demonstrate that a non-aggregating variant of MIP-1α, BB-10010, introduced before the radiation treatment and extending for several days thereafter, results in recovery that can better withstand subsequent cycles of cytotoxic treatment.

### Materials and methods

### *Mice*

Male B6D2F1 (C57Bl♀×DBA2♂) mice, aged 10 weeks at the start of experiments, were used throughout and all procedures were carried out under licence from the Home Office, Animals (Scientific Procedures) Act, 1986.

### *BB-10010/MIP-1α*

BB-10010 was kindly supplied by British Biotech Pharmaceuticals Ltd. (Oxford, UK) as a non-aggregating, genetically engineered variant of human MIP-1α (or LD78). It was shown to have greatly increased solubility compared with human MIP-1α and was equipotent with MIP-1α in receptor binding, calcium mobilisation, inhibition of colony formation and

Correspondence: BI Lord
Received 17 January 1996; revised 1 April 1996; accepted 9 May 1996

thymidine suicide assays (Hunter et al., 1995). It is prepared to standards required for clinical use with purity better than 98% and the protein contains less than 2 endotoxin units $mg^{-1}$ as determined by the limulus amoebocyte lysate assay (Hunter et al., 1995). BB-10010 was administered by mini-osmotic pumps (Alzet 2001, CA, USA) implanted subcutaneously on the backs of mice and delivering a uniform BB-10010 dose of 40 μg per 24 h period for 7 days.

### CFU-S assays

Haemopoietic spleen colony-forming units (CFU-S) were assayed as previously described (Lord, 1993). Briefly, mice (groups of 20) were irradiated (whole body) with 15.25 Gy $^{60}Co$ γ-rays (0.95 Gy $h^{-1}$). They were then injected intravenously with 0.2 ml of a freshly prepared suspension of bone marrow cells from mice treated as described above. Eight days (ten mice) and 12 days (ten mice) later the recipient mice were killed. Their spleens were excised, fixed and the macroscopic colonies counted using a dissecting microscope.

### Marrow repopulating ability assays (MRA)

The MRA was measured as the generation of 12 days CFU-S during 13 days' growth in the marrow by an extension of the CFU-S assay (see Lord, 1993 for details), on day 14 of the third and fourth treatment cycles. An extra five irradiated (primary) recipients were injected with the bone marrow suspension. After 13 days their femora were removed. Bone marrow suspensions were made and assayed for CFU-$S_{12}$ in secondary groups of ten irradiated recipients as described above. MRA was calculated as:

$$c \times p \times q \text{ per femur} \qquad \frac{c \times p \times q}{N} \times 10^5 \text{ per } 10^5 \text{ cells,}$$

where $N$ = number of donor marrow cells per femur; $c$ = number of CFU-$S_{12}$ colonies per secondary spleen; $1/q$ = fraction of donor marrow cells injected to primary recipient; and $1/p$ = fraction of primary recipient marrow injected in secondary recipient.

Three secondary assays were carried out for each primary recipient bone marrow pool for which $p = 10$, 20 and 40. In these experiments $q = 50$ or 100.

### Protocols

(1) Groups of three mice were implanted subcutaneously with mini-osmotic pumps delivering BB-10010 (40 μg per 24 h period for 7 days) or its vehicle, phosphate-buffered saline (PBS). Three to four hours later they were exposed (whole body) to 4.5 Gy γ-rays from a caesium-137 source (dose rate 2.5 Gy $min^{-1}$) and after 7 days the spent pumps were removed. Groups of mice were killed at 1, 7 and 14 days after irradiation and their femora and spleens removed. Cell suspensions were made in Fischer's medium from the bone marrow and spleen (Lord 1993 for details), counted and assayed for day 8 (CFU-$S_8$) and day 12 (CFU-$S_{12}$) colony-forming units. The cellular concentrations of the inoculation suspensions were adjusted so that 0.2 ml injected contained the fraction of a femur or spleen indicated in Table I. These figures should be considered merely as a guide. Throughout the course of the four experiments reported below, they were continuously refined to optimise the average spleen colony counts, where possible, at about 10.

This 2 week cycle of pump, irradiation and assays was repeated three more times in a total observation period of 56 days. On days 42 and 56 (ends of the third and fourth treatment cycles) marrow cells were additionally assayed for MRA.

(2) A separate series of experiments was conducted to explore whether BB-10010 has any direct radioprotective capacity or whether its presence is important only during the recovery phase. Observations were limited in these experiments to two 14 day cycles of 4.5 Gy whole body $^{137}Cs$-γ irradiation and recovery. Groups of three mice were implanted with minipumps dispensing BB-10010 (or PBS) from day 0 (3–4 h before irradiation) to 7, from 1 day after irradiation to 8 days or from 7 days after irradiation to 14 days in each treatment cycle. They were killed on day 14 of the second treatment cycle and bone marrow CFU-S assays carried out as before.

**Table I** Fractions of one femur or spleen injected for CFU-S assay

| Assay day[a] | BM | Spleen | MIP-BM | MIP-spleen |
|---|---|---|---|---|
| 1 | 1/3 | 1/6 | 1/3 | 1/6 |
| 7 | 1/10 | 1/10 | 1/40 | 1/40 |
| 14 | 1/100 | 1/100 | 1/100 | 1/100 |

[a]Repeated for each 14 day cycle of treatment and assay.

### Results

The repeated irradiation treatment regimen was conduct in four separate experiments and the overall results are shown in Table II and Figures 1 and 3. For each cycle of treatment, the cellularity of the control bone marrows fell to about 20–30% of normal within 24 h but recovered to near normal levels within 14 days (Table II). A similar pattern of loss and recovery was evident in the BB-10010-treated mice and at the end of each of the four treatment cycles, the average marrow cellularity was 29% higher than in the controls ($P = 0.002$). In the first treatment cycle, bone marrow CFU-S were reduced to less than 1% of their normal levels and in 14 days, CFU-$S_8$ and CFU-$S_{12}$ recovered to 40% and 20% respectively (Figure 1). BB-10010 induced a small, but non-significant increase in the recovery rate of both CFU-$S_8$ and CFU-$S_{12}$.

Repeated cycles of irradiation increased the damage to CFU-S in the untreated marrows. One day after irradiation, CFU-S survival levels were successively lower (Figures 1 and 2) as were the 14 day recovery levels (Figures 1 and 3), CFU-$S_8$ reaching only 10% and CFU-$S_{12}$ 6% of their

**Table II** Femoral bone marrow cellularity in mice subjected to repeated cycles of sublethal irradiation, with or without BB-10010

| Time (days) | Femur ($\times 10^{-6}$) | Femur ($\times 10^{-6}$) with BB-10010 |
|---|---|---|
| 0 | 20 | 20 |
| 1 | 5.1±0.5 | 4.2±0.4 |
| 7 | 15.8±2.0 | 20.0±2.5 |
| 14 | 17.0±1.7 | 18.1±2.3 |
| 15 | 7.2±1.6 | 8.2±5.6 |
| 21 | 23.2±3.1 | 20.0±3.4 |
| 28 | 16.5±2.1 | 23.3±2.6 |
| 29 | 4.0±1.4 | 4.1±0.7 |
| 35 | 13.4±2.7 | 24.5±4.1 |
| 42 | 16.0±2.4 | 18.9±1.6 |
| 43 | 4.1±0.3 | 5.0±0.4 |
| 49 | 20.8±3.5 | 14.0±2.8 |
| 56 | 14.7±2.5 | 22.3±3.1 |

Mice were exposed to 4.5 Gy γ-rays on day 0 and at 14 day intervals thereafter. Data are for three to four experiments±s.e. Data for day 0 are standardised norms for these mice and are presented simply as approximate reference points.

**Figure 1** Eight and twelve day CFU-S in the bone marrow of mice subjected to repeated 14 day cycles of total body, 4.5 Gy $\gamma$ irradiation. The shaded bars on the abscissae indicate the presence of a mini-osmotic pump dispensing BB-10010 (●) or PBS (○). Data are means from 3–4 experiments ± s.e. Data for day 0 are standardised norms for these mice and are presented simply as reference points.

**Figure 2** Detail of the 1 day after irradiation nadirs in bone marrow CFU-S following sequential 2-weekly doses of 4.5 Gy $\gamma$-rays (whole body). (○) Irradiation + PBS; (●) irradiation + BB-10010.

**Figure 3** Detail of the 14 day after irradiation recovery values for bone marrow CFU-S following sequential 2-weekly doses of 4.5 Gy γ-rays (whole body). (○) Irradiation + PBS; (●) irradiation + BB-10010.

starting levels after the fourth cycle of treatment. Intrinsic errors in measuring the very low CFU-S numbers 1 day after irradiation are unavoidably high. Figure 2, however, indicates that BB-10010 had little effect in the first cycle, but after the fourth cycle of treatment, about twice as many CFU-S survived (Figures 1 and 2 for CFU-$S_8$ and CFU-$S_{12}$, $P<0.01$ in the fourth cycle). The degree of recovery was maintained with each successive treatment cycle (Figures 1 and 3, $P<0.001$ over cycles 2–4 for CFU-$S_8$ and CFU-$S_{12}$) and at the end of the fourth treatment cycle both CFU-$S_8$ and CFU-$S_{12}$ were about 35% of normal levels in the BB-10010-treated mice compared with less than 10% without BB-10010.

At the end of each treatment cycle, splenic CFU-$S_8$ had recovered equally well with or without BB-10010 but for the most part, maximum recovery was achieved earlier (by 7 days) with BB-10010 (Figure 4). This was true also for CFU-$S_{12}$ which in the non-protected mice were still on the recovery curve at 14 days in the second, third and fourth cycles.

Marrow repopulating ability is normally recorded for these mice at about $10^5$ per femur or 500 per $10^5$ marrow cells (Lord and Woolford, 1993). Thirteen day recovery marrow in the third and fourth cycles of sublethal irradiation yielded an MRA of only 6500–7300 per femur at concentrations less than 10% of normal (Table III). Treatment with BB-10010 yielded an MRA of 26 600 ($P<0.001$) after the third cycle and 11 206 ($P<0.02$) after the fourth cycle of irradiation (Table III). The corresponding increase in cellular concentration of MRA cells of 1.3–3 times reflected the increase previously reported in self-renewal capacity of the stem cell populations following MIP-1α treatment (Lord, 1995).

Over two cycles of irradiation treatment, CFU-S in mice receiving PBS alone recovered to 730 per femur (Table IV). Pumps delivering BB-10010 during and after irradiation increased this to 1270 ($P=0.01$). Less advantage was obtained when BB-10010 dosing was delayed by 1 day (960 CFU-S per femur, *NS*), and none at all when dosing in the second half of each radiation cycle.

### Discussion

It is a common feature associated with repeated cycles of cytotoxic therapies that accumulating damage to the haemopoietic tissue often results in delays to the administration of the later treatments; indeed possibly to the curtailment of further cycles. Experimentally, it is known that repeated doses of sublethal irradiation also result in accumulating damage to the spleen colony-forming units (Hendry *et al.*, 1974). We chose, therefore, to use irradiation as a convenient model to test the potential of BB-10010 to maintain recovery of the stem cell populations. The 4 × 4.5 Gy irradiation model described by Hendry *et al.* (1974) was adopted, although the repeat interval was shortened to 14 days. This was to ensure suboptimally recovered haemopoietic tissue and, therefore, avoid any likelihood of masking beneficial effects of BB-10010. The progressively lower 1 day survivals and 14 day recovery peaks with increasing cycle number confirm that haemopoietic damage was accumulated through the experiment (Figures 2 and 3). In addition, and in spite of their somewhat greater radioresistance (Meijne *et al.*, 1991; Ploemacher *et al.*, 1992), the marrow repopulating cells also demonstrated significant accrued damage (Table II).

It has previously been shown that protection from hydroxyurea damage, using MIP-1α, results in a more rapid recovery of the multipotent progenitor populations that are relatively enriched in the better self-renewing cells (Lord, 1995) – probably the cells with MRA. This result complemented those of Verfaille *et al.* (1994) who demon-

**Figure 4** Eight and twelve day CFU-S in the spleens of mice subjected to repeated 14 day cycles of total body, 4.5 Gy γ-irradiation. The shaded bars on the abscissae indicate the presence of a mini-osmotic pump dispensing BB-10010 (●) or PBS (○). Data are means from 3–4 experiments ± s.e. Data for day 0 are standardised norms for these mice and are presented simply as reference points.

**Table III** MRA of progenitor cells in bone marrow of mice subjected to repeated cycles of sublethal irradiation, with or without BB-10010

| *Day* | *Bone marrow donor* | *Cells per donor femur* ($\times 10^{-6}$) | *CFU-S*$_{12}$ *primary femur* (=c×p) | *MRA Per donor femur* (=c×p×q) |
|---|---|---|---|---|
| 0 | Normal | 20 | – | $10^5$ |
| 42 | Irradiated | 13.5 | 65.3 ± 3.5 | 6533 ± 352 |
| (*n* = 3) | Irradiated + BB-10010 | 17.75 | 266 ± 14 | 26600 ± 1386 |
| 56 | Irradiated | 15.75 ± 0.05 | 61.0 ± 4.6 | 7266 ± 688 |
| (*n* = 9) | Irradiated + BB-10010 | 22.0 ± 3.2 | 114 ± 19 | 11206 ± 1313 |

Three secondary assays were carried out for each primary recipient bone marrow pool (taken at 13 days after transplant) for which $p = 10$, 20 and 40. In these experiments $q = 50$ or 100. *c*, *p* and *q* are defined in the text. [a]Data for normal mice are taken from Lord and Woolford (1993). Current data are mean values ± s.e.

**Table IV** Recovery of bone marrow CFU-S following radiation: dosing with BB-10010 at vaious times before or after radiation

| *BB-10010 treatment* | *CFU-S per femur*[a] |
|---|---|
| 4.5 Gy γ-rays only | 730 ± 94 |
| Days 0–7 | 1270 ± 127 |
| Days 1–8 | 960 ± 69 |
| Days 7–14 | 690 ± 64 |

[a]Data for CFU-S$_8$ and CFU-S$_{12}$ combined. Results are for mean of three experiments ± s.e.

strated that the equivalent *in vitro* long-term culture-initiating cells were maintained better in cultures treated with MIP-1α (in combination with IL-3). This modulation in favour of self-renewal potential effectively reduces differentiation potential. It does not necessarily adversely affect the production of differentiated cells, however. Indeed, the increased reserves of 'stem' cells generated by increased self-renewal, can provide a pool that is more than adequate to compensate the reduced differentiation rate. We reasoned, therefore, that recovery from sublethal doses of irradiation could be similarly enhanced by the presence of MIP-1α, during and after the period of irradiation.

The results presented here indicate that this rationale was sound. BB-10010 gave little, if any, protection from the first dose of irradiation but the better recovery characteristics, particularly in respect of the highly enriched MRA, ensured that the second and subsequent irradiations caused progressively less initial damage (Figure 2) and that the recovery patterns did not significantly deteriorate (Figure 3).

In these experiments, our primary aim was to assess the ability of BB-10010 to support a better quality haemopoietic progenitor cell component in the bone marrow and spleen. The design of the experiments, therefore, was such that simultaneous recording of peripheral blood cell levels was not practical. Preliminary experiments (unpublished data) with cyclophosphamide and 5-fluorouracil (5-FU) suggest that neutrophil production is promoted by 1–2 days, supporting the observations of Dunlop *et al.* (1992). These experiments demonstrated a fuller and earlier recovery of the progenitor populations with full restoration of the haemopoietic, nucleated cell populations and consistent with conditions for earlier recovery of the mature cell products. The very early recovery of the splenic CFU-S population (Figure 4) would be expected to contribute to such a process. However, the role of the spleen is not clear. It has to be recognised that, relative to total haemopoiesis, the splenic contribution is small and, in a comparable human protocol, would probably play no part.

In these experiments, BB-10010 was supplied continuously for 7 days of each cycle. At this stage, we have no evidence that this length of time is necessary, nor even that the MIP-1α remains active under these conditions for the whole 7 days. The timing and administration protocols, therefore, remain the subject of further investigation.

While it is clear that this type of protocol is a practical scenario, the mechanisms by which MIP-1α is promoting recovery are not clear. The demonstration that MIP-1α contributes to a better maintenance of primitive haemopoietic stem cells (Verfaillie *et al.*, 1994), that a CFU-S population regenerating after exposure to MIP-1α has a higher self-renewal capacity (Lord, 1995) and supported by a recent observation that recovery is enhanced following cyclophosphamide (a non-S-phase-specific cytotoxic agent) treatment (Parker *et al.*, 1995), all suggest that recovery kinetics are improved by MIP-1α. It is not clear whether MIP-1α (BB-10010 in this instance) has any direct radioprotective effect. Initial depopulation of CFU-S (1 day nadir, cycle 1, Figure 2) suggests not. However, those recovery characteristics were clearly improved by the presence of BB-10010 at the time of irradiation (Table IV). Delay of exposure to BB-10010 until 1 day after irradiation ameliorated the recovery significantly and when administered during the later stages of recovery, it gave no beneficial advantage (Table IV).

In summary, we conclude that the cumulative effects of non-cycle-specific cytotoxic therapies on normal haemopoiesis can be ameliorated by the administration of BB-10010/MIP-1α during, and in the aftermath of, such treatments. These experiments should, therefore, provide a base for protection experiments and treatments with other clinically appropriate cyclical therapies using cytotoxic alkylating agents. Ultimately, they should be directly transferable to appropriate clinical scenarios in the treatment of cancer.

**Acknowledgements**
The authors wish to thank the Cancer Research Campaign for their continued support of this work and British Biotech Pharmaceuticals Ltd. for support and provision *ad libitum* of BB-10010/MIP-1α.

### References

CLEMENTS JM, CRAIG S, GEARING AJH, HUNTER MG, HEYWORTH CM, DEXTER TM AND LORD BI. (1992). Biological and structural properties of MIP-1α expressed in yeast. *Cytokine*, **4**, 76–82.

COOPER S, MANTEL C AND BROXMEYER HE. (1994). Myelosuppressive effects *in vivo* with very low dosages of monomeric recombinant murine macrophage inflammatory protein-1α. *Exp. Hematol.*, **22**, 186–193.

DUNLOP DJ, WRIGHT EG, LORIMORE S, GRAHAM GJ, HOLYOAKE T, KERR DJ, WOLPE SD AND PRAGNELL IB. (1992). Demonstration of stem cell inhibition and myeloproliferative effects of SCI/rh MIP-1α *in vivo*. *Blood*, **79**, 2221–2225.

GRAHAM GJ, WRIGHT EG, HEWICK R, WOLPE SD, WILKIE NM, DONALDSON D, LORIMORE S AND PRAGNELL IB. (1990). Identification and characterisation of an inhibitor of haemopoietic stem cell proliferation. *Nature*, **344**, 442–444.

HENDRY JH AND LAJTHA LG. (1972). The response of hemopoietic colony-forming units to repeated doses of X-rays. *Radiat. Res.*, **52**, 309–315.

HENDRY JH, TESTA NG AND LAJTHA LG. (1974). Effect of repeated doses of X-rays or 14 MeV neutrons on mouse bone marrow. *Radiat. Res.*, **59**, 645–652.

HUNTER MG, BAWDEN L, BROTHERTON D, CRAIG S, CRIBBES S, CZAPLEWSKI LG, DEXTER TM, DRUMMOND AH, GEARING AH, HEYWORTH CM, LORD BI, MCCOURT M, VARLEY PG, WOOD LM, EDWARDS RM AND LEWIS PJ. (1995). BB-10010: an active variant of human macrophage inflammatory protein-1α with improved pharmaceutical properties. *Blood*, **86**, 4400–4408.

LORD BI. (1993). *In vivo* assays for multipotential and marrow repopulating cells. In *Haemopoiesis, A Practical Approach*. Testa NG, Molineux G (eds). pp. – 120 Press at Oxford University Press: Oxford, New York, Tokyo.

LORD BI. (1995). MIP-1α increases the self-renewal capacity of the hemopoietic spleen-colony-forming cell population *in vivo*. *Growth Factors*, **12**, 145–149.

LORD BI AND WOOLFORD LB. (1993). Proliferation of spleen colony forming units ($CFU-S_8$, $CFU-S_{13}$) and cells with marrow repopulating ability. *Stem Cells*, **11**, 212–217.

LORD BI, MORI KJ, WRIGHT EG AND LAJTHA LG. (1976). An inhibitor of stem cell proliferation in normal bone marrow. *Br. J. Haematol.*, **34**, 441–445.

LORD BI, LIU FL, POJDA Z AND SPOONCER E. (1987). Inhibitor of haemopoietic CFU-S proliferation: assays, production sources and regulatory mechanisms. In *The Inhibitors of Hematopoiesis*, vol. 162. Najman A, Guigon M, Gorin N-C, Mary J-Y (eds). pp. 227–239. INSERM/John Libbey Eurotext: London.

LORD BI, DEXTER TM, CLEMENTS JM, HUNTER MG AND GEARING AJH. (1992). Macrophage inflammatory protein protects multipotent hematopoietic cells from the cytotoxic effects of hydroxyurea *in vivo*. *Blood*, **79**, 2605–2609.

MEIJNE EIM, VAN DER WINDEN-GROENEWEGEN RJM, PLOEMACHER RE, VOS O, DAVID JAG AND HUISCAMP R. (1991). The effects of X-irradiation on hematopoietic stem cell compartments in the mouse. *Exp. Hematol.*, **19**, 617–623.

PARKER AN, SIM AR, GRAHAM GJ, CLARK SC AND PRAGNELL IB. (1995). Macrophage inflammatory protein-1α has a positive effect on bone marrow recovery kinetics following treatment with cyclophosphamides (abstract). *Exp. Hematol.*, **23**, 465, 874P.

PLOEMACHER RE, VAN OS RP, VAN BEURDEN CAJ AND DOWN JD. (1992). Murine hemopoietic stem cells with long-term engraftment and marrow repopulating ability are less radiosensitive to gamma irradiation than are spleen colony forming cells. *Int. J. Radiat. Biol.*, **61**, 489–499.

TILL JE AND MCCULLOCH EA. (1961). A direct measurement of the radiation sensitivity of normal mouse bone marrow cells. *Radiat. Res.*, **14**, 213–222.

VERFAILLIE CM, CATANZARRO PM AND LI WN. (1994). Marcophage-inflammatory protein 1 alpha, interleukin-3 and diffusible marrow stromal factors maintain human hematopoietic stem cells for at least eight weeks *in vitro*. *J. Exp. Med.*, **179**, 643–649.

WRIGHT EG AND LORD BI. (1977). Regulation of CFU-S proliferation by locally produced and endogenous factors. *Biomed. Exp.*, **27**, 215–218.


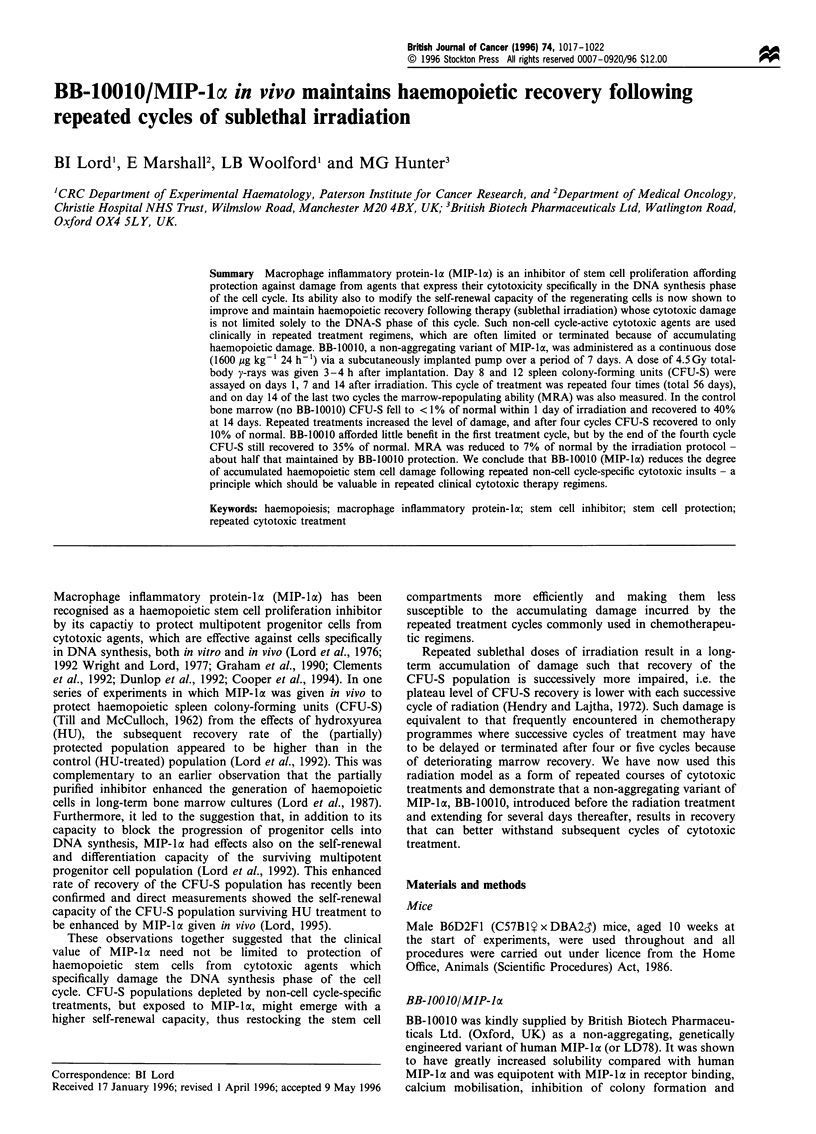

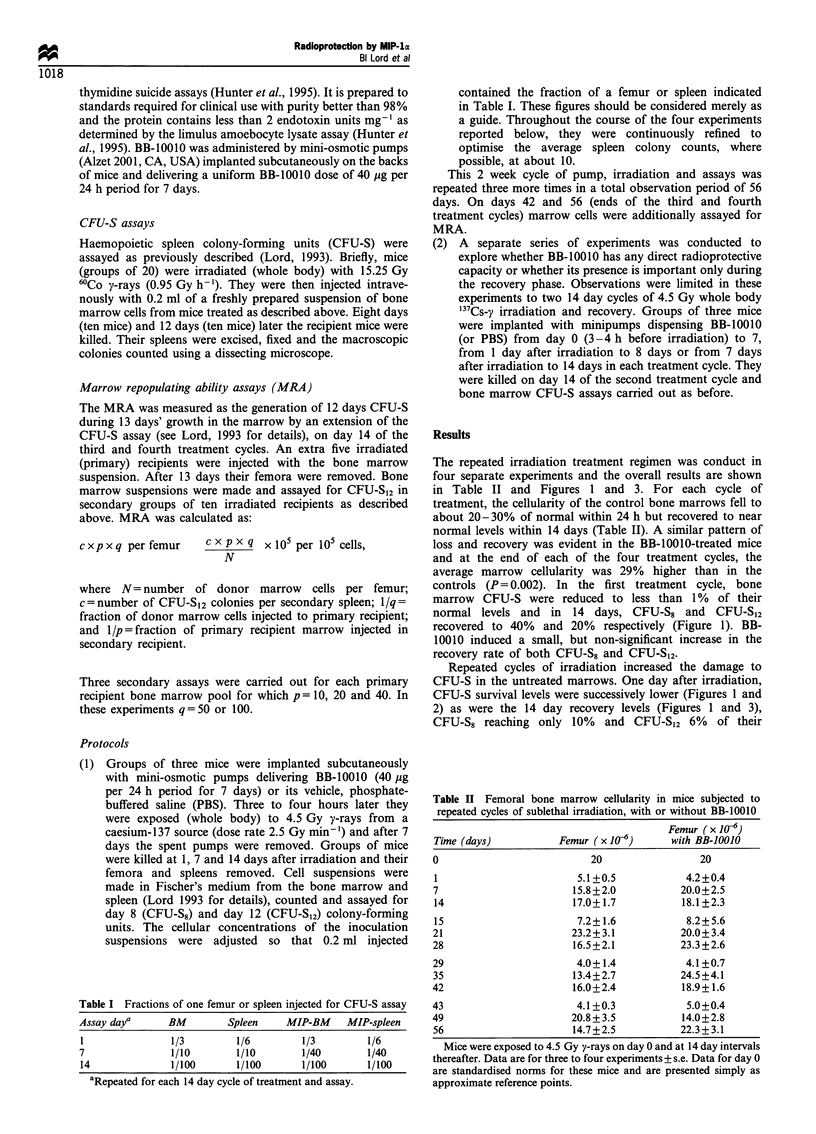

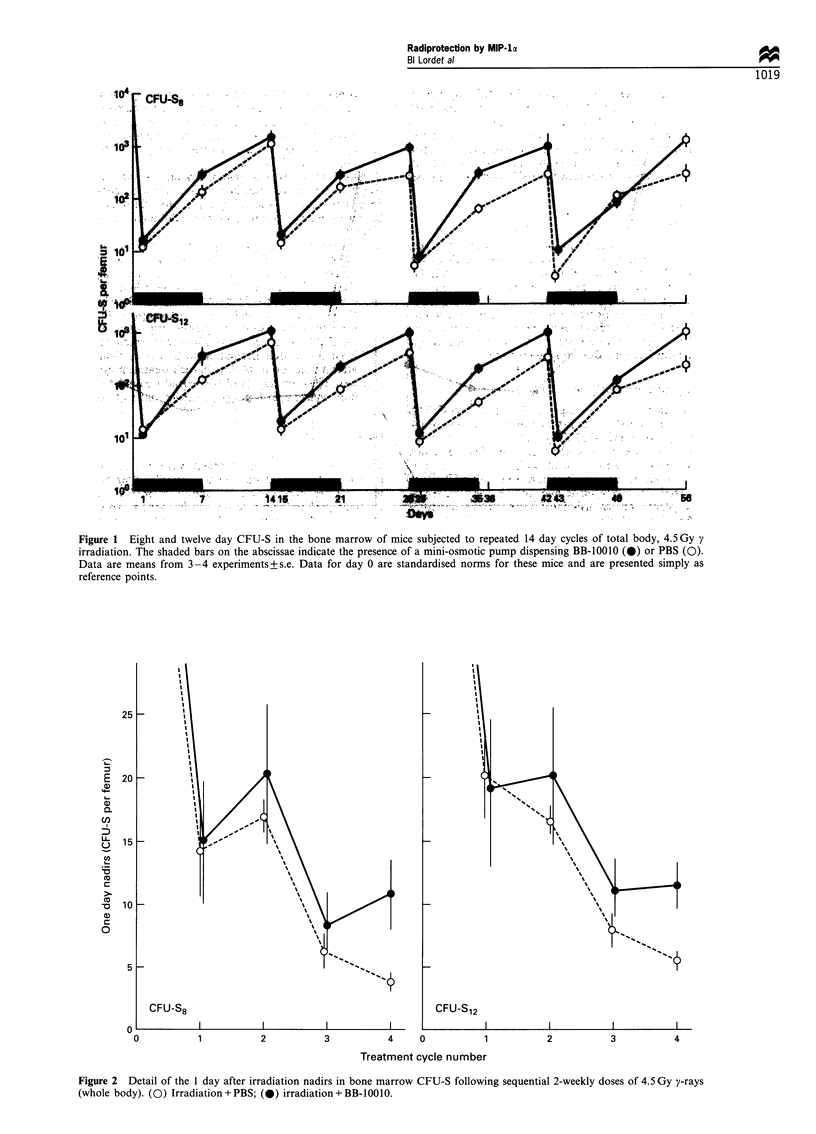

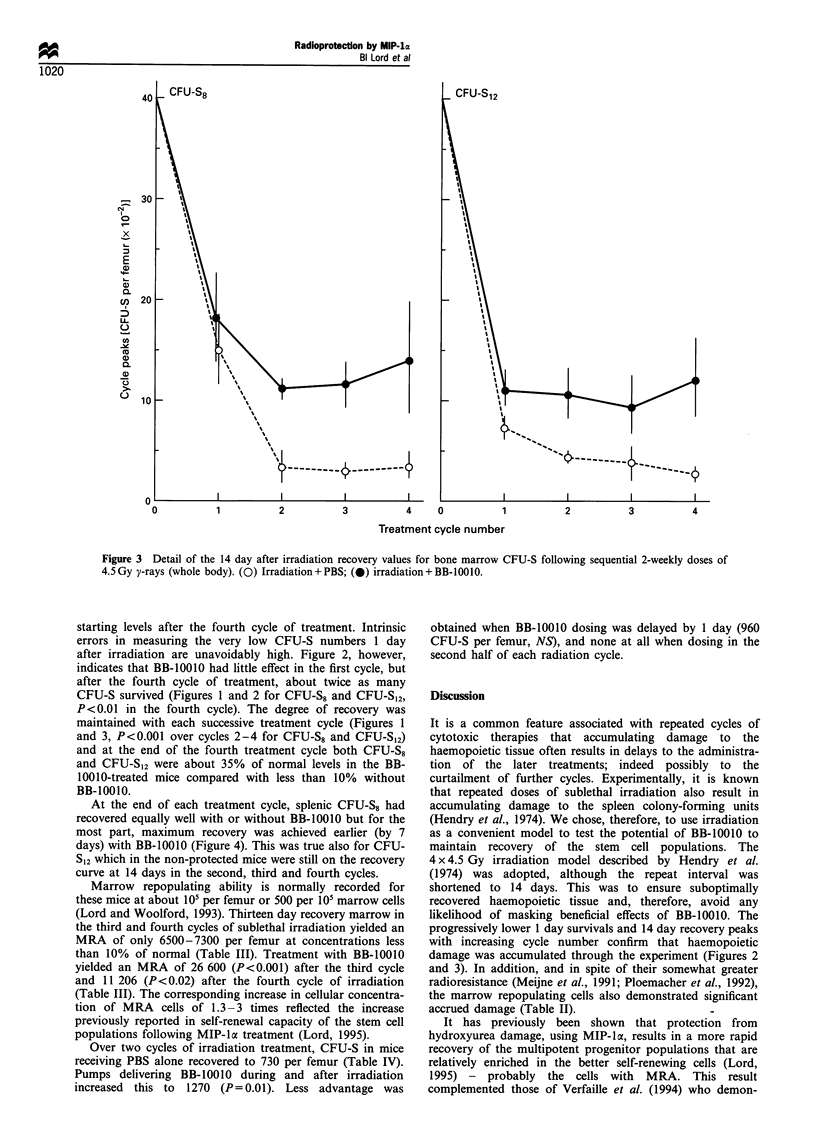

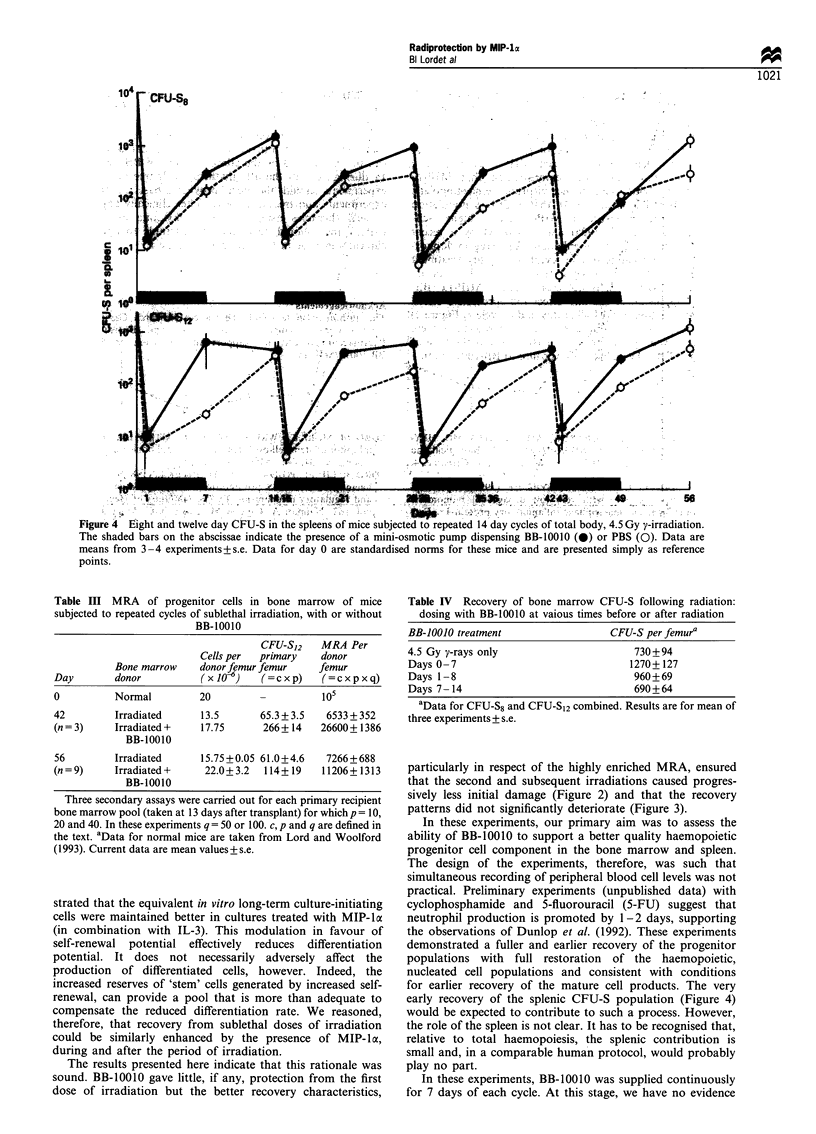

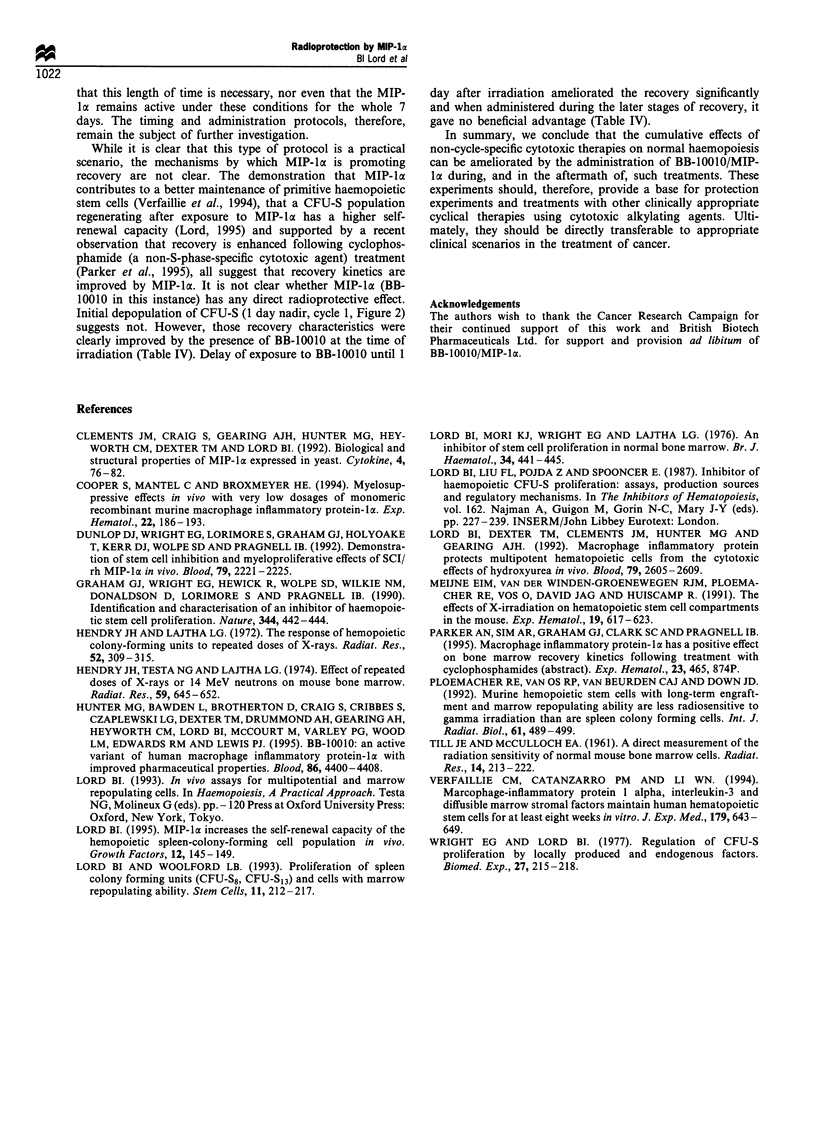


## References

[OCR_00621] Clements J. M., Craig S., Gearing A. J., Hunter M. G., Heyworth C. M., Dexter T. M., Lord B. I. (1992). Biological and structural properties of MIP-1 alpha expressed in yeast.. Cytokine.

[OCR_00625] Cooper S., Mantel C., Broxmeyer H. E. (1994). Myelosuppressive effects in vivo with very low dosages of monomeric recombinant murine macrophage inflammatory protein-1 alpha.. Exp Hematol.

[OCR_00631] Dunlop D. J., Wright E. G., Lorimore S., Graham G. J., Holyoake T., Kerr D. J., Wolpe S. D., Pragnell I. B. (1992). Demonstration of stem cell inhibition and myeloprotective effects of SCI/rhMIP1 alpha in vivo.. Blood.

[OCR_00639] Graham G. J., Wright E. G., Hewick R., Wolpe S. D., Wilkie N. M., Donaldson D., Lorimore S., Pragnell I. B. (1990). Identification and characterization of an inhibitor of haemopoietic stem cell proliferation.. Nature.

[OCR_00645] Hendry J. H., Lajtha L. G. (1972). The response of hemopoietic colony-forming units to repeated doses of x-rays.. Radiat Res.

[OCR_00650] Hendry J. H., Testa N. G., Lajtha L. G. (1974). Effect of repeated doses of x-rays or 14 MeV neutrons on mouse bone marrow.. Radiat Res.

[OCR_00653] Hunter M. G., Bawden L., Brotherton D., Craig S., Cribbes S., Czaplewski L. G., Dexter T. M., Drummond A. H., Gearing A. H., Heyworth C. M. (1995). BB-10010: an active variant of human macrophage inflammatory protein-1 alpha with improved pharmaceutical properties.. Blood.

[OCR_00691] Lord B. I., Dexter T. M., Clements J. M., Hunter M. A., Gearing A. J. (1992). Macrophage-inflammatory protein protects multipotent hematopoietic cells from the cytotoxic effects of hydroxyurea in vivo.. Blood.

[OCR_00669] Lord B. I. (1995). MIP-1 alpha increases the self-renewal capacity of the hemopoietic spleen-colony-forming cells following hydroxyurea treatment in vivo.. Growth Factors.

[OCR_00679] Lord B. I., Mori K. J., Wright E. G., Lajtha L. G. (1976). Inhibitor of stem cell proliferation in normal bone marrow.. Br J Haematol.

[OCR_00674] Lord B. I., Woolford L. B. (1993). Proliferation of spleen colony forming units (CFU-S8, CFU-S13) and cells with marrow repopulating ability.. Stem Cells.

[OCR_00698] Meijne E. I., van der Winden-van Groenewegen R. J., Ploemacher R. E., Vos O., David J. A., Huiskamp R. (1991). The effects of x-irradiation on hematopoietic stem cell compartments in the mouse.. Exp Hematol.

[OCR_00709] Ploemacher R. E., van Os R., van Beurden C. A., Down J. D. (1992). Murine haemopoietic stem cells with long-term engraftment and marrow repopulating ability are more resistant to gamma-radiation than are spleen colony forming cells.. Int J Radiat Biol.

[OCR_00716] TILL J. E., McCULLOCH E. A. (1961). A direct measurement of the radiation sensitivity of normal mouse bone marrow cells.. Radiat Res.

[OCR_00719] Verfaillie C. M., Catanzarro P. M., Li W. N. (1994). Macrophage inflammatory protein 1 alpha, interleukin 3 and diffusible marrow stromal factors maintain human hematopoietic stem cells for at least eight weeks in vitro.. J Exp Med.

[OCR_00726] Wright E. G., Lord B. I. (1977). Regulation of CFU-S proliferation by locally produced endogenous factors.. Biomedicine.

